# Psychometrics and validation of the EQ-5D-5L instrument in individuals with ischemic stroke in Lithuania

**DOI:** 10.3389/fpsyg.2023.1284859

**Published:** 2023-12-06

**Authors:** Saulius Taroza, Julius Burkauskas, Narseta Mickuviene, Nijole Kazukauskiene, Aurelija Podlipskyte

**Affiliations:** Laboratory of Behavioral Medicine, Neuroscience Institute, Lithuanian University of Health Sciences, Palanga, Lithuania

**Keywords:** psychometrics, quality of life, ischemic stroke, depression, cross-sectional studies, Lithuanian people, anxiety

## Abstract

**Background:**

Experiencing stroke is associated with deterioration in health-related quality of life (HRQL). One of the generic tools used for HRQL assessment is the EuroQol instrument of five dimensions and five levels (EQ-5D-5L), which has not yet been validated in Lithuania. This study aimed to evaluate validity, reliability, and factor structure of the EQ-5D-5L instrument in a sample of Lithuanian individuals at the end of the first week after experiencing ischemic stroke (IS).

**Methods:**

The study had a cross-sectional design, including 134 individuals [61.9% men and 38.1% women; median (IQR) age was 66 years (59–73) years, in the final analysis]. Alongside the EQ-5D-5L, psychological distress was evaluated using the Hospital Anxiety and Depression Scale (HADS), Patient Health Questionnaire-9 (PHQ-9), and Generalized Anxiety Disorder Assessment-7 (GAD-7); neurological impairment with the National Institutes of Health Stroke Scale (NIHSS); and functional independence with the Barthel index (BI). Confirmatory factor analysis (CFA) was performed for validation of the factor structure.

**Results:**

The internal consistency of the EQ-5D-5L instrument was 0.81. A significant ceiling effect (17.2%) of the descriptive part of the EQ-5D-5L was detected. The convergent validity of the EQ-5D-5L descriptive system was confirmed, with significant correlations with the other scales used, except for the visual analog scale. The two-factor (“physical” and “emotional”) model was confirmed by CFA, with acceptable fit [root mean square error of approximation (RMSEA) = 0.045, RMSEA 90% CI = 0.000–0.145; comparative fit indices (CFI) = 0.996; non-normal fit index (NFI) = 0.983; Tucker–Lewis Index (TLI) = 0.936; χ^2^/*df* = 1.27)].

**Conclusion:**

This study provides information on the psychometric properties of the EQ-5D-5L instrument in Lithuanian individuals, showing that the EQ-5D-5L descriptive system is a reliable and valid tool for HRQL assessment. The Lithuanian version of the descriptive part of the EQ-5D-5L instrument is best expressed as a two-factor model, estimating the physical and emotional dimensions of HRQL in individuals who have experienced IS.

## 1 Introduction

HRQL is recognized as of paramount importance in health outcomes (Kaplan, 1990; Bunevicius et al., 2022). In one study, a single question about self-rated health has been shown to be strongly associated with mortality at follow-up (DeSalvo et al., 2006). Although there are some problems arising with the universal definition of HRQL (Karimi and Brazier, 2016), it is usually described as the daily level of functioning and perceived health-associated wellbeing on a personal level (Stenman et al., 2010). Therefore, the multifaceted construct of HRQL is characterized subjectively by an individual as the impact of illness and its treatment on physical, mental, and social domains of functioning (Revicki et al., 2014). It is assumed that the evaluation of HRQL enables better patient-directed healthcare than the traditional biomedical model, which is focused primarily on diagnosis and treatment (Kaplan, 2003).

Although there are many HRQL instruments implemented in practice, according to previous research, there is no “best” or “worst” instrument (Coons et al., 2000); the choice should depend on the purpose of the measurement. The attractiveness of each instrument depends on the ease of use, its psychometric properties, free availability, and usefulness in the economic assessment of public health interventions. One such instrument belongs to one of the most widely used generic methods of HRQL assessment—the EQ-5D set of instruments (Pequeno et al., 2020). The latest version of the EQ-5D for adults is the EQ-5D-5L, which has better psychometric properties (increased reliability and sensitivity) than its precedent, the EQ-5D-3L (Feng et al., 2021). Regarding the psychometric properties of the EQ-5D-5L, this instrument is valid and reliable for health status assessment across a broad spectrum of populations, with acceptable responsiveness. However, it has some limitations, including the tendency for a ceiling effect and the lack of positive health aspects (Feng et al., 2021).

According to the EQ-5D-5L factor structure, at least one study has suggested that it consists of two latent factors encompassing physical and psychological functioning (Gao et al., 2019), but other studies suggested one-factor structure (Bilbao et al., 2022). However, some concern has recently been raised over the scale's lack of social dimension (Chen and Olsen, 2020). Despite the aforementioned limitations, this scale is used widely due to its simplicity, free-of-charge use for non-commercial reasons, availability in many languages, and applicability for various conditions (Lau et al., 2022).

Stroke, as one of the most frequent worldwide causes of disability (Campbell and Khatri, 2020), is associated with reduced post-stroke HRQL (Cadilhac et al., 2010; Gall et al., 2010; Mar et al., 2015; Chen et al., 2019). In the United States, it has been shown that the consequences of stroke significantly impair the HRQL of respondents who are not committed to an institution compared with those without stroke (Xie et al., 2006). Another study, based on a population in northern Manhattan study, showed a significant worsening of HRQL independent of various risk factors, including functional independence, during the 5-year follow-up (Dhamoon et al., 2010). On the contrary, a study conducted in Lithuania with stroke survivors after 3 and 12 months using the 12-item Short Form Survey of Health showed that the survivors had poorer HRQL than the controls but showed remarkable improvement over time (Kranciukaite-Butylkiniene, 2014). Furthermore, hyperacute recanalization therapy in acute ischemic stroke (IS) is not clearly related to better long-term HRQL, despite better functional outcomes (Kainz et al., 2021). Based on the results of the mentioned studies, it is important to continue research on impaired post-stroke HRQL in order to better understand this phenomenon and thus make suggestions for HRQL improvement-directed interventions.

In terms of HRQL for stroke patients, the validity of the EQ-5D-5L instrument was recently demonstrated for individuals from Poland after stroke (Golicki et al., 2015). Another study performed in Taiwan proved the validity of this instrument for HRQL assessment in patients after stroke undergoing rehabilitation (Chen et al., 2016). Furthermore, a systematic review of the instruments for assessing self-reported HRQL after stroke showed that the EQ-5D instrument was the best choice (Cameron and Wales, 2022).

Given that the EQ-5D-5L has not been validated in Lithuania, this study focused on the psychometric properties, including applicability, internal consistency, validity, and factor structure of this instrument in Lithuanian residents who had experienced IS.

## 2 Materials and methods

### 2.1 Study procedure

This study was a part of a research described previously (Burkauskas et al., 2014). Individuals who had experienced acute IS and were admitted to the three different Lithuanian health institutions (Klaipeda University Hospital, Hospital of the Lithuanian University of Health Sciences Kauno Klinikos, and Klaipeda Seamen's Hospital) were invited by a neurologist in the emergency room on duty to participate in the study during two 1-year periods, starting in 2013 and 2016, respectively. In total, 612 consecutive individuals were asked to participate in this study.

The inclusion criteria were: (1) ages 18–80 years; (2) current diagnosis of acute IS as described by the World Health Organization criteria (Hatano, 1976), affirmed by neurovisual imaging with brain-computer or magnetic resonance tomography; and (3) capable of communication and cognition, according to a Mini-Mental State Exam (MMSE) score of more than 19, assessed at the end of the first week. The exclusion criteria were: (1) co-diagnosis of severe pathology (infection, liver and/or renal insufficiency, and malignancy); (2) noted thyroidopathy and/or intake of thyroid-affecting substances; and (3) arrival 2 days later after the onset of IS.

The following characteristics of the individuals were assessed in the emergency department: (1) age; (2) sex; (3) body mass index; (4) presence of premorbid disability, defined as dependency in daily activity according to a Modified Rankin Scale (mRS) score of ≥3; (5) use of antithrombotic drugs; (6) chemical thrombolysis; and (7) stroke risk factors, including arterial hypertension, atrial fibrillation, smoking, diabetes mellitus, previous cerebral ischemic event, and experienced myocardial infarction. In addition, the individuals' neurological impairment was assessed using the National Institutes of Health Stroke Scale (NIHSS) (Spilker et al., 1997).

At the end of their hospital stay, all study individuals were asked to fill out questionnaires in paper form: (1) EQ-5D-5L (Herdman et al., 2011) for HRQL and (2) Hospital Anxiety and Depression Scale (HADS) (Zigmond and Snaith, 1983), Patient Health Questionnaire-9 (PHQ-9) (Kroenke et al., 2001), and Generalized Anxiety Disorder assessment-7 (GAD-7) (Spitzer et al., 2006) for psychological distress assessment. At this time point after IS, individuals were checked for functional independence according to the Barthel index (BI) (Mahoney and Barthel, 1965). At the end of the first year of the study, participants were asked to fill in the EQ-5D-5L once more.

**Figure 1:**
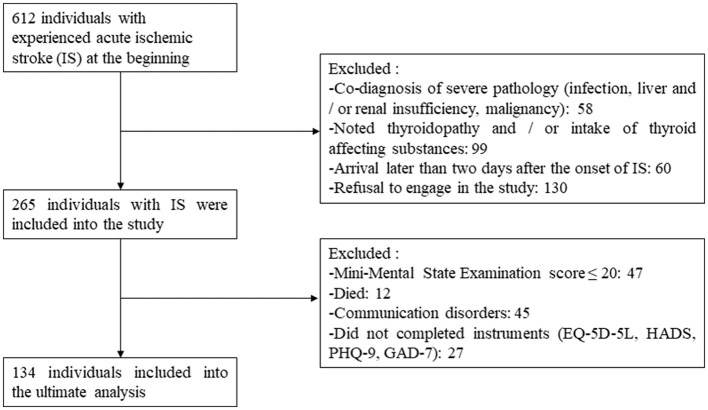
Recruitment process.

For a sufficient sample, power analysis was based on the suggested rule—at least 10 respondents to 1 scale item (Boateng et al., 2018), and it was more than 50 individuals in our case. Figure 1 shows the selection of individuals for the study. In total, 134 individuals were included in the final analysis.

The study was conducted in accordance with the Declaration of Helsinki and met the requirements of the Regional Biomedical Research Ethics Committee, with the assigned licenses P1-BE-2-11/2013 and P2-BE-2-11/2013. Individuals were included only after giving written consent for participation in this study.

### 2.2 Measurements and applied questionnaires

#### 2.2.1 Modified rankin scale

This global disability-assessing instrument is used to evaluate dependence in daily life activities among individuals with experienced stroke. Despite the fact that this scale is weighted more toward physical disability, it captures (indirectly) other attributes essential to daily activity, including wellbeing, socialization, mood, and cognitive status. The estimate of mRS ranges from 0 (no disability at all) to 6 (dead). The reliability of this scale, including inter-rater and test–retest, lies within moderate and strong limits, respectively (Banks and Marotta, 2007). This scale shortage is associated with its low stroke specificity because it automatically includes other disability causes, such as a previous bone fracture (Kasner, 2006).

#### 2.2.2 National institutes of health stroke scale

The NIHSS scale for quantification of stroke-related neurological impairment consists of 11 neurological examination categories, scored from 0 to 4, with a total score from 0 to 42 (Spilker et al., 1997). On this scale, a higher score indicates more pronounced neurological impairment. The reliability of this scale lies within reasonable limits (Lyden, 2017). To use this scale, one needs special training to reach sufficient reliability and validity. Another shortage of this scale is its inappropriateness for self-report usage or by telephone (Kasner, 2006).

#### 2.2.3 Mini-mental state exam

MMSE is a screening tool used to evaluate cognitive functioning including its five domains (orientation, memory, attention, recollection, and language), with a score rating from 0 to 30 (Folstein et al., 1975). This scale is characterized by better sensitivity for capturing moderate and higher cognitive impairment than mild cognitive impairment (Tombaugh and McIntyre, 1992).

#### 2.2.4 Barthel index

BI was created for the assessment of independence in activities of daily living (Mahoney and Barthel, 1965). This instrument is composed of 10 items, with four possible choices scored as 0, 5, 10, or 15. The possible scores range from 0 to 100. Higher values indicate better functional independence. The reliability of this scale was 0.98 when assessed with Cronbach's α (Shinar et al., 1987). The limitation of this scale is its “ceiling effect” because it does not include many aspects that are important for daily activity, such as emotional disturbances, cognition, and language among others (Kasner, 2006).

#### 2.2.5 EQ-5D-5L

The EQ-5D-5L instrument is composed of two parts, including a descriptive part made up of five different health dimensions (mobility, self-care, usual activities, pain/discomfort, and anxiety/depression) with five possible options and a thermometer-like visual analog scale (EQ-VAS) numbered from 0 (“worst” HRQL) to 100 (“best” HRQL) to measure overall health (Herdman et al., 2011). The self-described descriptive part of the EQ-5D-5L can be expressed as one of 3,125 different health states, from “No” (“best” HRQL or level 1) to “Extreme” (“worst” HRQL or level 5) problems in all dimensions, or expressed as one index value (EQ index), ranging from slightly <0–1, with higher values indicating a better HRQL. The EQ index mirrors how positive or negative the health state is, depending on the preferences of the country of study. The EQ-VAS is scored by the respondent marking “X” on the scale and separately clarifying the marked point with a number indicating their current health. The self-filled paper version of the EQ-5D-5L in the Lithuanian language has been available since 2014. The self-complete version of the Lithuanian EQ-5D-5L paper was used with formal consent from EuroQol Group with the assigned number 53563. As there is no calculated country-specific EQ index value set for the Lithuanian population, the set from the closest available country is selected, which is from the German population (Ludwig et al., 2018). In the current study, Cronbach's alpha for the measurement was 0.81, while McDonald's Omega was 0.83.

#### 2.2.6 Hospital anxiety and depression scale

The HADS, a self-report screening scale, is composed of two parts, assigned to depression and anxiety severity assessment, respectively (Zigmond and Snaith, 1983). Each part has seven items with four possible options ranging from 0 to 3 according to the psychological distress experienced during the past week, thus generating a score from 0 to 21, with a higher score indicating more pronounced psychological distress. According to previous studies, Cronbach's α of the anxiety part varied from 0.68 to 0.83, while the depression part ranged from 0.67 to 0.90 (Bjelland et al., 2002). The shortcoming of HADS is its dependence on self-reporting which could be impaired because of language and emotional disturbances. A validated Lithuanian version of this instrument (Bunevicius, 1991) was used with permission from the “GL Education Group”.

#### 2.2.7 Patient health questionnaire-9

The PHQ-9, a self-report questionnaire for estimating the severity of depression, is composed of nine questions, each of them reflecting depression symptoms in the past 2 weeks, rated from 0 to 3 (Kroenke et al., 2001). The total score can range from 0 to 27, with higher scores reflecting more severe depression. At the end of the questionnaire, there is an additional optional question for global functional impairment assessment. Recently, this scale was validated in a Lithuanian student sample and individuals with anxiety and mood disorders with an estimated reliability (Cronbach's α) of 0.86 (Pranckeviciene et al., 2022; Stanyte et al., 2023). Currently, the Lithuanian version is available on the screener's website (https://www.phqscreeners.com/select-screener). The limitation of this scale is its dependence on intact respondents' communication.

#### 2.2.8 Generalized anxiety disorder-7

The GAD-7 questionnaire was developed for generalized anxiety screening and assessment of its severity (Spitzer et al., 2006). This instrument is composed of seven questions, reflecting anxiety symptoms during the past 2 weeks, with four possible answers ranging from “not at all” to “nearly every day”, scored 0 and 3, respectively. Thus, the overall GAD-7 score can range from 0 to 21, with a higher score showing more pronounced symptoms of anxiety. Recently, in Lithuania, the GAD-7 was validated as a first-line anxiety screening tool (Pranckeviciene et al., 2022; Stanyte et al., 2023). For this instrument, Cronbach's α was 0.91. Lithuanian form of instrument is available on the website (https://www.phqscreeners.com/select-screener). The limitation of this scale is its dependence on intact respondents' communication.

### 2.3 Statistical analysis

Statistical analysis was performed using IBM SPSS Statistics for Windows (version 28) (SPSS Inc, Chicago, IL, USA) and IBM SPSS AMOS 28 (IBM Corp., Armonk, NY, USA). Quantitative data were expressed as the mean (±standard deviation, SD) or median (interquartile range, IQR), with normality checked using the Kolmogorov–Smirnov test. Qualitative data were expressed in number (%).

The reliability of the used questionnaires is expressed as Cronbach's α and McDonald's omega (Hayes and Coutts, 2020). Cronbach's α coefficient estimates between 0.70 and 0.95 were considered to be acceptable (Tavakol and Dennick, 2011). Both the ceiling and floor effects of the EQ-5D-5L health profile, with scores of level “1” or “5” in all dimensions, EQ index, EQ-VAS, HADS for depression and anxiety, HADS total, PHQ-9, and GAD-7 scores, were reported as the proportion of individuals reporting the highest and lowest possible estimates, respectively. A questionnaire was considered to show a ceiling or floor effect if at least 15% of respondents scored the highest or lowest achievable score (Terwee et al., 2007).

The convergent evidence for the EQ-5D-5L, including the separate dimensions of this scale, the EQ index and the EQ-VAS, with other used self-reported questionnaires (BI and NIHSS), was evaluated using Spearman's correlation coefficient. The closeness of co-variation was defined according to the value of the correlation coefficient: ≤ 0.30 as negligible, 0.31–0.50 as low, 0.51–0.70 as moderate, 0.71–0.90 as high, and 0.91–1.00 as very high (Mukaka, 2012).

Confirmatory factor analysis was performed for validation of the factor structure considered for one (Bilbao et al., 2022) and two factors (Santiago et al., 2021). Analysis of Moment Structures (AMOS) 27.0 software was used to test the model of the EQ-5D-5L using CFA. The proposed thresholds for the CFA fit indices were: CFI > 0.90 adequate and >0.95 good; TLI > 0.90 adequate and >0.95 good; NFI > 0.90 adequate and >0.95 good; RMSEA < 0.08; and χ^2^/*df* with the desired range of 2–5 (Hooper et al., 2008; Brown, 2015). In addition, standardized coefficients for each EQ-5D-5L item were calculated.

The dimensions of the EQ-5D-5L in stroke patients at baseline and after a year were compared using the Wilcoxon signed-rank test. Changes were interpreted according to the Pareto Classification of Health Change (Devlin et al., 2010).

## 3 Results

Table 1 shows the basic characteristics of study participants. Table 2 shows the main identified characteristics of the used scales. Estimates of the reliability coefficient were within acceptable limits for all scales, except for the HADS depression scale, for which this was marginal (Cronbach's α = 0.699). The ceiling effect of the EQ-5D-5L health profile with a full health state of “11111” was highlighted at a significant level in 17.2% of all respondents. In contrast, no floor effect was detected (health state of “55555”). Regarding the EQ index, alongside the same ceiling estimate for the EQ-5D-5L health profile, the floor effect was observed in 0.7% of all respondents. Ceiling and floor effects of the EQ-VAS were observed in 0.7 and 1.5% of individuals, respectively. Of the other scales for the evaluation of psychological distress, only the GAD-7 showed a significant ceiling effect, with a fixed estimate of 41.7%. A significant floor effect was observed for the BI (23.5%).

**Table 1:** Characteristics of all study patients.

	**Total group**
Sample size	134
**Demographics**	
Age, years median (IQR)	66.0 (58.8–73.0)
Age, years mean (SD)	67 (9.6)
Sex, M, *n* (%)	83 (61.9)
Sex, F, *n* (%)	51 (38.1)
Body mass index, median (IQR)	27.7 (24.8–31.8)
Premorbid disability, *n* (%)	6 (3.8)
Used antithrombotic drugs, *n* (%)	50 (37.3)
Chemical thrombolysis, *n* (%)	40 (32.8)
**Vascular risk factors**	
Arterial hypertension, *n* (%)	100 (74.6)
Atrial fibrillation, *n* (%)	42 (31.3)
Smoking, *n* (%)	32 (23.9)
Diabetes mellitus, *n* (%)	20 (14.9)
Previous cerebral ischemic event, *n* (%)	23 (17.2)
Previous myocardial infarction, *n* (%)	12 (9.0)

F, female; M, male; IQR, interquartile range; NIHSS, National Institutes of Health Stroke Scale.

**Table 2:** Characteristics of the scales used in the study population (*n* = 134).

**Measures**	**No. of items**	**Mean ±SD**	**Median (IQR)**	**Min**	**Max**	**Ceiling, *n* (%)**	**Floor, *n* (%)**	**Cronbach's α**
**Quality of life**								
EQ-5D-5L	5							
Mobility		2.55 ± 1.47	2 (1–4)	1	5	45 (33.6)	21 (15.7)	
Self-care		2.14 ± 1.43	1 (1–3)	1	5	69 (51.5)	14 (10.4)	
Usual activities		2.51 ± 1.47	2 (1–4)	1	5	48 (35.8)	20 (14.9)	
Pain/discomfort		1.99 ± 1.12	2 (1–3)	1	5	62 (46.3)	4 (3.0)	
Anxiety/depression		1.73 ± 1.02	1 (1–2)	1	5	75 (56.0)	4 (0.3)	
EQ-5D-5L total		10.92 ± 4.94	10 (7–15)	5	25	23 (17.2)	0 (0.0)	0.809
EQ index		0.69 ± 0.32	0.82 (0.47–0.93)	−0.34	1	23 (17.2)	1 (0.7)	
EQ VAS		58.36 ± 23.81	60 (50–80)	0	100	1 (0.7)	2 (1.5)	
**Psychological distress**								
HADS								
HADS depression	7	4.87 ± 3.70	4 (2–7)	0	16	7 (5.2)	1 (0.7)	0.699
HADS anxiety	7	4.79 ± 3.70	4 (2–7)	0	18	12 (8.9)	1 (0.7)	0.751
HADS total	14	9.66 ± 6.14	8 (6–14)	0	33	2 (1.5)	1 (0.7)	0.781
PHQ-9	9	5.18 ± 4.89	4 (2–7)	0	21	14 (11.2)	3 (2.4)	0.796
GAD-7	7	2.95 ± 3.44	2 (0–5)	0	14	35 (41.7)	3 (3.6)	0.826
**Functional independence**								
Barthel index	10	69.4 ± 32.79	80 (50–95)	0	100	48 (25.4)	13 (6.9)	0.949
**Neurological impairment**								
NIHSS	11	8.94 ± 6.98	7 (4–12)	0	39	6 (2.9)	1 (0.5)	0.805

VAS, visual analog scale; HADS, Hospital Anxiety and Depression Scale; PHQ-9, Patient Health Questionnaire 9; GAD-7, Generalized Anxiety Disorder assessment-7; NIHSS, National Institutes of Health Stroke Scale; SD, standard deviation.

The convergent validity of the EQ-5D-5L was analyzed, and its correlation with other variables is presented in Table 3. A positive but low correlation was established between the EQ-5D-5L mobility dimension and HADS depression (*r* = 0.337, *p* < 0.001), HADS total (*r* = 0.328, *p* < 0.001), GAD-7 (*r* = 0.300, *p* = 0.006), and NIHSS (*r* = 0.413, *p* < 0.001); between the EQ-5D-5L self-care dimension and NIHSS (*r* = 0.483, *p* < 0.001); between the EQ-5D-5L usual activity dimension and HADS total (*r* = 0.306, *p* < 0.001) and NIHSS (*r* = 0.472, *p* < 0.001); between the EQ-5D-5L pain/discomfort dimension and PHQ-9 (*r* = 0.410, *p* < 0.001) and GAD-7 (*r* = 0.312, *p* = 0.004); and between the EQ-5D-5L anxiety/depression dimension and HADS depression (*r* = 0.393, *p* < 0.001), HADS anxiety (*r* = 0.438, *p* < 0.001), HADS total (*r* = 0.495, *p* < 0.001), PHQ-9 (*r* = 0.338, *p* < 0.001), and GAD-7 (*r* = 0.338, *p* < 0.001). A statistically significant (*p* < 0.001), moderate, negative correlation was found between the EQ-5D-5L mobility (*r* = −0.695, *p* < 0.001) and usual activity dimensions (*r* = −0.663, *p* < 0.001), and a high negative correlation was found between the self-care dimension (*r* = −0.756, *p* < 0.001) and the BI. The correlation between the EQ index and HADS depression (*r* = −0.405, *p* < 0.001), HADS anxiety (*r* = −0.339, *p* < 0.001), HADS total (*r* = −0.443, *p* < 0.001), PHQ-9 (*r* = −0.411, *p* < 0.001), GAD-7 (*r* = −0.392, *p* < 0.001), and NIHSS (*r* = −0.371, *p* < 0.001) was low and negative but positive and moderate with BI (*r* = 0.612, *p* < 0.001). The correlations between other variables were at a negligible correlation level and/or statistically insignificant.

**Table 3:** Convergent evidence of the EQ-5D-5L with HADS, PHQ-9, GAD-7, NIHSS, and Barthel Index in the overall sample (*n* = 134).

**Scales**	**EQ-5D-5L**						
	**Mobility**	**Self-care**	**Usual activities**	**Pain/discomfort**	**Anxiety/ depression**	**EQ index**	**EQ VAS score**
**HADS**							
HADS depression	0.337^**^	0.228^*^	0.249^*^	0.220^*^	0.393^**^	−0.405^**^	−0.194
HADS anxiety	0.216^*^	0.172^*^	0.264^*^	0.226^*^	0.438^**^	−0.339^**^	−0.159
HADS total	0.328^**^	0.237^*^	0.306^**^	0.266^*^	0.495^**^	−0.443^**^	−0.210^*^
PHQ-9	0.270^*^	0.255^*^	0.244^*^	0.410^**^	0.338^**^	−0.411^**^	−0.144
GAD-7	0.300^*^	0.160	0.284^*^	0.312^*^	0.350^*^	−0.392^*^	−0.202
NIHSS	0.413^**^	0.483^**^	0.472^**^	0.006	0.112	−0.371^**^	−0.071
Barthel Index	−0.695^**^	−0.756^**^	−0.663^**^	−0.139	−0.112	0.612^**^	0.209^*^

VAS, a visual analog scale; HADS, Hospital Anxiety and Depression Scale; PHQ-9, Patient health questionnaire-9; GAD-7, Generalized anxiety disorder assessment-7; NIHSS, National Institutes of Health Stroke Scale.

^*^p < 0.05.

^**^p < 0.001.

Table 4 shows that the fit of the unidimensional structure was mixed since RMSEA (>0.08) had unacceptable values. On the other hand, the fit of the two-dimensional structure was excellent since both CFI (>0.95) and RMSEA (<0.08) had good values. The two-factor model showed an acceptable fit (RMSEA = 0.045, 90% CI = 0.000–0.145; CFI = 0.996; NFI = 0.983; TLI = 0.991; χ^2^/*df* = 1.27).

**Table 4:** Confirmatory factor analysis of two measurement models of EQ-5D-5L.

	**χ^2^/*df***	**CFI**	**TLI**	**NFI**	**RMSEA (90% CI)**
1-factor model	2.87	0.968	0.936	0.953	0.119 (0.049–0.193)
2-factor model	1.27	0.996	0.936	0.983	0.045 (0.000–0.145)

CFI, comparative fit index; TLI, Tucker–Lewis Index; NFI, non-normal fit index; RMSEA, root-mean-square error of approximation; 90% CI, 90% confidence interval of the RMSEA.

The results supporting convergent evidence between isolated factors from the EQ-5D-5L and other applied measures are presented in Table 5, expressed as correlations. Factor 1 (physical) was positively and significantly (0.201–0.377, *p* < 0.05) correlated with the HADS depression, HADS total, PHQ-9, and NIHSS within low correlation limits but negatively (−0.708, *p* < 0.001) and highly correlated with BI. Additionally, a positive, low correlation (0.362–0.478, *p* < 0.001) was established between factor 2 (emotional) and HADS anxiety, HADS depression, PHQ-9, and GAD-7, and a high correlation was established with HADS total (*p* < 0.001). Standardized coefficients for EQ-5D-5L items ranged from 0.55 (anxiety/depression), 0.62 (pain discomfort), 0.82 (mobility), and 0.87 (activities) to 0.92 (self-care).

**Table 5:** Convergent evidence of the factors of EQ-5D-5L with HADS, PHQ-9, GAD-7, NIHSS, and Barthel Index in the overall sample.

	**EQ-5D-5L**	
	**Factor 1**	**Factor 2**
**HADS**		
HADS depression	0.371^**^	0.362^**^
HADS anxiety	0.201^*^	0.478^**^
HADS total	0.350^**^	0.505^**^
PHQ-9	0.301^**^	0.449^**^
GAD-7	0.271^*^	0.451^**^
NIHSS	0.377^**^	−0.016^*^
Barthel Index	−0.708^**^	−0.098^*^

HADS, Hospital Anxiety and Depression Scale; PHQ-9, Patient health questionnaire-9; GAD-7, Generalized anxiety disorder assessment-7; NIHSS, National Institutes of Health Stroke Scale.

^*^p < 0.05.

^**^p < 0.001.

After 1 year of IS, the EQ-5D-5L data were available for 117 of the included individuals. The comparison of EQ-5D-5L dimensions between two different time points is shown in Table 6. Significant changes were observed in the mobility (*p* = 0.013) and anxiety/depression (*p* < 0.001) dimensions.

**Table 6:** Descriptive statistics of the EQ-5D-5L dimensions in stroke patients at baseline and after a year.

**Dimension**	**Baseline, median (IQR)**	**Follow-up, median (IQR)**	***p***
	***N*** = **117**		
Mobility	2.55 ± 1.47	2.26 ± 1.34	**0.013**
Self-care	2.11 ± 1.40	2.08± 1.43	0.795
Usual activities	2.49 ± 1.45	2.43 ± 1.53	0.695
Pain/discomfort	1.97 ± 1.11	2.07 ± 1.19	0.443
Anxiety/depression	1.72 ± 1.01	2.19 ± 1.30	**< 0.001**

Bolded, *p* < 0.05.

## 4 Discussion

Our results indicate that the descriptive EQ-5D-5L system could be used as a reliable and valid tool for HRQL assessment in individuals living in Lithuania during their hospitalization period due to IS. Furthermore, the presented results suggest the presence of two EQ-5D-5L factors in individuals who have experienced IS.

This study establishes that the EQ-5D-5L health profile shows no floor effect but has a significant ceiling effect. This is consistent with the ceiling effect described in the post-stroke population in Taiwan, which was even higher (20%) (Chen et al., 2016). Another study, which included native Polish speakers, found a much lower ceiling effect of 5.6% (Golicki et al., 2015). In general, it is agreed that the EQ-5D-5L is prone to a large ceiling effect because of its nature in measuring more aspects of negative health than positive health (Feng et al., 2021). In addition, the tendency for more positive HRQL self-evaluation in Lithuania may be associated with cultural and historical (post-Soviet) aspects, such as denial of psychological distress (Gailiene, 2021). In terms of the EQ index and EQ-VAS, the ceiling and floor effects were non-significant.

The convergent validity of the EQ-5D-5L health profile justifies the identified significant correlations between the anxiety/depression dimension and all other used scales for measuring psychological distress, as well as between the pain/discomfort dimension and PHQ-9 and GAD-7. EQ-5D-5L dimensions such as mobility, self-care, and usual activities correlated more with scales that included a mobility component, namely, the NIHSS scale, and even with BI. In addition, the latter EQ-5D-5L dimensions were correlated with the HADS total, and the mobility dimension was correlated with HADS depression and GAD-7. The EQ index showed a significant correlation with all included instrument scores, adding additional justification for the convergent validity of the descriptive EQ-5D-5L system. As for EQ-VAS, no significant correlations point to unjustified convergent validity of this EQ-5D-5L component. An established difference could be attributed to the EQ-5D-5L health profile and the EQ index to social perspectives and EQ-VAS to personal perspectives. Furthermore, another explanations could be that the EQ-VAS is a wider construct than the EQ-5D-5L health profile; misinterpretation of the EQ-VAS filling instructions; and difficulty in understanding this two-pole scale (Feng et al., 2014), especially keeping in mind that our study population consisted of individuals with an organically injured brain—the substrate for cognition. In addition, the study from Taiwan did not show EQ-VAS power for predicting rehabilitation outcomes after stroke (Kainz et al., 2021).

Our study revealed the existence of two factors of the EQ-5D-5L, which is in line with a study exploring the validity of this instrument among individuals with heart disease (Gao et al., 2019). The latter study separated only the EQ-5D-5L anxiety/depression dimension into the second factor, while our results additionally identified the pain/discomfort dimension. In our study, we highlighted that the first factor, composed of EQ-5D-5L mobility, self-care, and usual activity dimensions, could be attributed to the physical component of this scale, while the other two dimensions—pain/discomfort and anxiety/depression—could be attributed to the emotional one. According to our results, research from Australia with indigenous people found identical EQ-5D-5L two-dimensional latent factor composition (Santiago et al., 2021). On the other hand, in Spain, evaluating psychometrics of this instrument in individuals with depression showed uni-dimensionality of latent factors (Bilbao et al., 2022). Different results could be attributed to different clinical entities (stroke, strictly organic brain disease with physical and emotional consequences, vs. depression, with a more pronounced emotional component).

Convergent analysis of revealed factors substantiated their relevance, with a significant correlation between the first factor and NIHSS and BI and between the second factor and applied questionnaires dedicated to psychological distress assessment (HADS, PHQ-9, and GAD-7). Here, the low positive correlation between NIHSS and the first factor could be attributed to differences in the evaluation of NIHSS and EQ-5D-5L in time and less sensitivity of the latter measure to neurologic deficits evaluated with NIHSS such as neglect and visual disturbances (van der Ende et al., 2023).

Finally, our results showed that HRQL was not static after stroke. An unadjusted analysis of the EQ-5D-5L health profile confirmed meaningful changes in responses to the mobility and anxiety/depression dimensions. Here, mobility improved, but anxiety/depression deteriorated.

## 5 Strengths, limitations, and applications

The strengths of the present study are the participation of three different centers, a large enough sample size, and the use of validated scales. The limitations of the study were the exclusion of individuals with communication disorders, the unavailability of radiological data related to stroke volume and place, and the lack of comparisons made with other HRQL instruments. This study further expands the territory of usage of the EQ-5D-5L instrument for HRQL assessment in individuals after IS, adding the country of Lithuania. This validated instrument creates an opportunity for further clinical and economic research dedicated to improving IS-associated HRQL in Lithuania.

## 6 Conclusion

This study adds knowledge of the psychometric properties of the EQ-5D-5L instrument in individuals who have experienced IS in Lithuania. The research confirmed that the EQ-5D-5L instrument and its derivative EQ index are a valid and reliable tool for HRQL assessment in individuals at the end of the first week after IS. In addition, the analysis revealed two factors behind the EQ-5D-5L health profile, with possible physical and emotional dimensions. The data did not support the validity of overall health expressed as EQ-VAS scoring in these individuals. Our study supports further research using the EQ-5D-5L instrument for HRQL assessment in individuals who have experienced stroke.

## Data availability statement

The raw data supporting the conclusions of this article will be made available by the authors, without undue reservation.

## Ethics statement

The studies involving humans were approved by Regional Biomedical Research Ethics Committee (Permission Numbers: BE-2-11/2013; P2-BE-2-11/2013). The studies were conducted in accordance with the local legislation and institutional requirements. The participants provided their written informed consent to participate in this study.

## Author contributions

ST: Investigation, Methodology, Writing—original draft. JB: Investigation, Methodology, Writing—review & editing. AP: Methodology, Writing—review & editing. NK: Project administration, Writing—review & editing. NM: Conceptualization, Funding acquisition, Resources, Writing—review & editing.
